# Clinical and Sociodemographic Characteristics of Cases Diagnosed with Autism Spectrum Disorder at the **Etlik City** Multidisciplinary Child and Adolescent Mental Health Center (ÇÖZGEM)

**DOI:** 10.5152/eurasianjmed.2025.24748

**Published:** 2025-08-11

**Authors:** Meryem Kaşak, Ayşegül Efe, Yusuf Selman Çelik, Şeyma Selcen Macit, Ülkü Beyza Gökmen

**Affiliations:** Department of Child and Adolescent Psychiatry, Ankara Etlik City Hospital, Ankara, Türkiye

**Keywords:** Autism spectrum disorder, early diagnosis, multidisciplinary care, sociodemographic factors

## Abstract

**Background:**

Autism spectrum disorder (ASD) is a neurodevelopmental condition marked by challenges in social interaction, communication, and the presence of restricted interests and repetitive behaviors. The increasing prevalence of ASD underscores the importance of early diagnosis and individualized interventions. This study investigates the sociodemographic and clinical characteristics of children aged 0-6 years diagnosed with ASD at the Etlik City Multidisciplinary Child and Adolescent Mental Health Center (ÇÖZGEM) and explores their healthcare journey from developmental delays to formal diagnosis.

**Methods:**

The medical records of 174 children evaluated at ÇÖZGEM between May and November 2024 were reviewed. Of these, 100 children diagnosed with ASD, with a mean age of 40.86 ± 16.92 months, were included. Multidisciplinary evaluations were conducted by specialists, including a child psychiatrist, clinical psychologist, and speech therapist.

**Results:**

Of the children diagnosed, 83% were boys and 17% were girls. Parents typically identified developmental concerns, focusing on language and social skill delays, at a mean age of 23.1 ± 10.94 months. The mean age at diagnosis was 31.54 ± 12.11 months, with an average delay of 8.44 ± 8.76 months between initial concern and diagnosis. After referral to ÇÖZGEM, the average waiting time for the first appointment was 18.21 ± 10.25 days, with diagnosis completed within 36.85 ± 19.8 days.

**Conclusion:**

This study highlights the importance of multidisciplinary teams in ASD diagnosis and intervention. Insights from ÇÖZGEM emphasize the need for parental awareness and streamlined healthcare pathways. Further studies with larger samples are necessary to validate these findings.

**Main Points**

- Autism spectrum disorder is a common neurodevelopmental condition.
- Early parental recognition is crucial for timely intervention.
- Cultural and systemic factors influence diagnostic delays.
- Individualized interventions improve outcomes for children with ASD.
- The multidisciplinary approaches applied at ÇÖZGEM significantly shorten the diagnostic process.

## Introduction

Autism spectrum disorder (ASD) is a complex neurodevelopmental condition marked by persistent challenges in social interaction, impaired communication skills, restricted interests, and repetitive behavioral patterns.^1^ The prevalence of autism, as reported by the Centers for Disease Control and Prevention (CDC) in the United States, has risen sharply over the years—from 1 in 110 children in 2006 to 1 in 69 in 2012, 1 in 44 in 2018, and most recently, 1 in 36 by 2020.^2^ The sharp increase in ASD diagnoses highlights the vital importance of early identification and the implementation of tailored early intervention programs, which are pivotal in optimizing developmental outcomes and family well-being.^3^

The early diagnosis of ASD facilitates access to medical and educational support during critical developmental periods, positively influencing developmental outcomes, alleviating family distress, and improving overall prognosis.^4^ The aim of diagnosis extends beyond identifying the condition; it also includes providing adequate support and guidance to the patient and their family. Routine screening for ASD is a cornerstone of pediatric care, particularly for children presenting with developmental delays, behavioral challenges, psychiatric symptoms, or genetic syndromes.^5^ The National Institute for Health and Care Excellence (NICE) guidelines emphasize that diagnostic evaluations for ASD should extend beyond merely confirming a diagnosis. The primary aim is to comprehensively assess an individual’s risks and physical, psychological, and social functioning to effectively determine their treatment and care needs and tailor appropriate support plans.^6^ The integration of multidisciplinary teams in ASD evaluations is strongly advocated by various guidelines,^7^^-^^9^ emphasizing their role in ensuring accurate diagnoses and formulating comprehensive therapeutic strategies tailored to individual needs. Integrating assessments of an individual’s symptoms, functional level, and interactions with their environment—conducted by professionals from various disciplines—is essential for accurate diagnosis, consistent follow-up, and multidimensional treatment planning.^10^ However, access to multidisciplinary teams or centers with experienced clinicians specializing in ASD remains limited globally, with such resources not uniformly available across all regions.^11^

To address these challenges, the Turkish Ministry of Health formalized the establishment of Multidisciplinary Child and Adolescent Mental Health Centers (ÇÖZGEM) through the regulation published in the Official Gazette on December 15, 2022, issue no. 32044.^12^ One of the first operational centers, “ Etlik City ÇÖZGEM,” completed its licensing process on March 27, 2024, under theAnkara Etlik City Hospital, Children’s Hospital, Child and Adolescent Psychiatry Clinic, and began offering formal healthcare services in May 2024. ÇÖZGEM was designed with the understanding that a multidisciplinary approach to the biopsychosocial diagnosis, follow-up, and treatment of mental health needs, particularly ASD, neurodevelopmental disorders, and developmental disabilities is both crucial and influential within the healthcare system. The multidisciplinary team at ÇÖZGEM includes professionals such as child and adolescent psychiatrists, individual service consultants (ISC), child development specialists, psychologists, speech and language therapists, occupational therapists, social workers, nurses, and medical secretaries, all working collaboratively to provide comprehensive care.

There are 11 licensed ÇÖZGEM centers across Türkiye. A literature review reveals no studies examining the characteristics of ASD cases referred to ÇÖZGEM. This study aims to explore the sociodemographic and clinical characteristics of children diagnosed with ASD at Ankara Etlik ÇÖZGEM while tracing their healthcare journey—from the initial recognition of developmental concerns to the final diagnosis. This study seeks to contribute to understanding the clinical characteristics of ASD in Türkiye and to provide a profile of service utilization within the ÇÖZGEM model of care.

The hypotheses of the study are (i) the age at which families first recognize ASD-related concerns, the age at which a formal diagnosis is made, and the duration of the diagnostic process at ÇÖZGEM are comparable to those reported in international multidisciplinary centers and (ii) higher CARS scores, indicating greater ASD severity, are associated with longer diagnostic timelines at ÇÖZGEM.

## Materials and Methods

### Study Design and Participants

**Figure 1.**
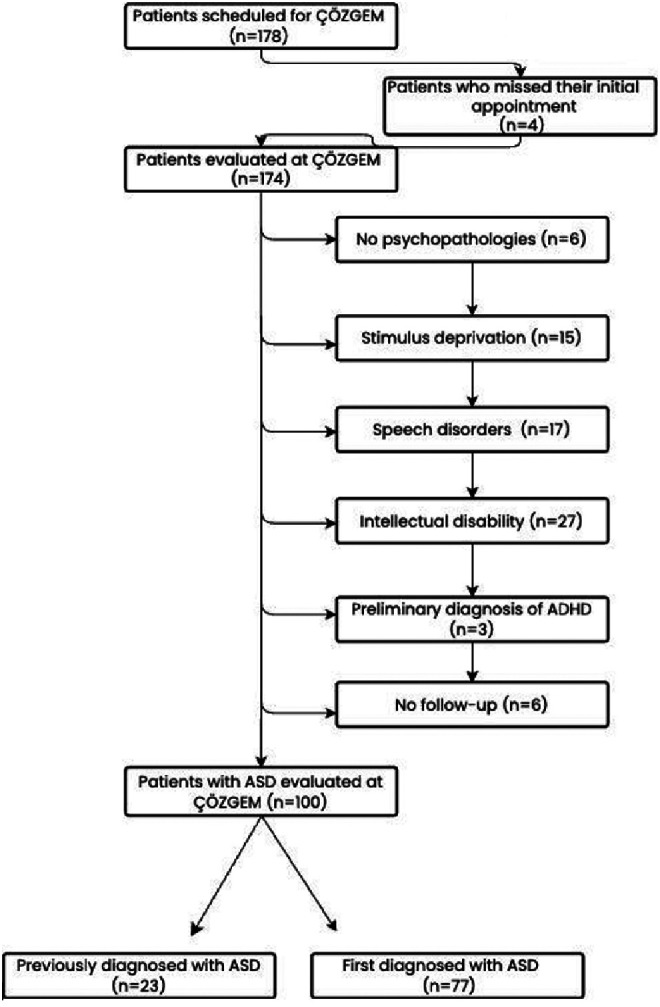
Flowchart of the study inclusion protocol.

This study included all cases referred to the Ankara Etlik City Hospital ÇÖZGEM Clinic that met the inclusion criteria between May 1, 2024, and November 1, 2024. Cases referred to ÇÖZGEM within the specified time frame were included if they had either received an ASD diagnosis during the evaluation process or had a pre-existing ASD diagnosis. Cases referred to ÇÖZGEM but subsequently diagnosed with conditions other than ASD were excluded from the study (Figure 1).

A total of 174 children (143 boys, 31 girls) with a mean age of 40.13 months (SD = 16.79; median = 37; range = 12-98 months) were evaluated. Among these, 100 children (83 boys and 17 girls) were diagnosed with ASD. The mean age of children diagnosed with ASD was calculated as 40.86 months (SD = 16.92; median = 38; range = 14-98 months). The ASD-diagnosed sample comprised children who had either received an ASD diagnosis prior to their ÇÖZGEM evaluation (n = 23) or were newly diagnosed during the evaluation process (n = 77).

Children, primarily aged 0-6 years (and those older than 6 years when clinically indicated), suspected of having ASD or other intellectual and developmental special needs, were referred to Etlik City ÇÖZGEM by physicians at all levels of general healthcare services. A medical referral form was utilized for patients from outside Ankara Etlik City Hospital. Although ÇÖZGEM primarily serves children aged 0-6 years, referrals for older children may occur when ethically justified. Two such cases over age 6 were diagnosed with ASD and retained in the sample to preserve data integrity.

### Diagnostic Process

Diagnostic evaluations and intervention planning were carried out by a multidisciplinary team including child and adolescent psychiatrists, ISC (unit nurse), child development specialists, clinical psychologists, speech and language therapists, social workers, occupational therapists, and nurses. Diagnoses were based on comprehensive developmental evaluations, including medical and psychosocial history, play-based observations, parent rating scales, and diagnostic tests. Weekly consensus meetings were held to integrate findings and ensure a holistic view of each case.

ÇÖZGEM follows a structured diagnostic workflow, where each specialist has standardized roles. The initial intake is conducted by the ISC, who collects detailed psychosocial and developmental history, clinical features, prior medical and psychiatric diagnoses, and information on family expectations and support systems. A child and adolescent psychiatrist confirms the diagnosis using DSM-5 criteria, clinical interviews, and the Childhood Autism Rating Scale (CARS).

Subsequently, referrals are made based on diagnostic needs. Psychometric developmental assessments are conducted using the Early Developmental Stages Inventory (EGE) and EGE-SE inventories, supported by clinical observations. Language skills and sensory profiles are assessed by speech therapists and occupational therapists, respectively. Meanwhile, the ISC coordinates diagnosis disclosure, psychoeducation, and interdisciplinary consultations. Social workers evaluate psychosocial support needs and facilitate access to legal rights. Data from interviews, assessments, and clinical observations are discussed during weekly team meetings to develop individualized short- and long-term rehabilitation plans. Additional medical evaluations (neurological, metabolic, genetic, audiometric, and visual) are arranged when needed. Sociodemographic and clinical data, psychiatric diagnoses, and comorbid conditions are recorded systematically. After diagnostic formulation, the team develops case-specific intervention plans within and beyond the unit. Follow-up is structured for at least 1 year and adjusted to each child’s individual needs.

### Ethical Considerations

The study was approved by the Ethics Committee of Ankara Etlik City Hospital (Date: 06.11.2024; Approval no: AEŞH-BADEK-2024-815), and general informed consent for research purposes was obtained from parents during the registration process.

### Psychometric Assessments

#### Sociodemographic, Medical, and Developmental History Data Form

Sociodemographic (age, gender, parental age, parental education level, parental occupation, family structure, number of siblings, and birth order), medical (perinatal/natal/postnatal history, psychiatric diagnosis and comorbidity status, medical diagnosis status), and developmental information (parents’ initial concerns, age at first visit to a healthcare facility, age at autism diagnosis, use of alternative treatments, special education process, etc.) about the child and their parents were collected. The ISC completed this form for all cases referred to ÇÖZGEM.

#### Childhood Autism Rating Scale 

Developed by Schopler et al. in 1980,^13^ the CARS is a behavioral assessment tool used to differentiate between ASD, intellectual disability, and typically developing children. A clinician completes the scale based on information obtained through interviews with the family, input from other relevant individuals, and direct observation of the child. The total CARS score ranges from 15 to 60, with scores between 30 and 36.5 indicating mild autism and scores of 37 and above indicating severe autism.

#### Early Developmental Stages Inventory

Early developmental stages inventory is a parent-completed screening tool designed to assess the development of children aged 4 to 60 months. It evaluates 5 domains: fine motor, gross motor, communication, problem-solving, and personal-social skills. Each domain includes 6 items scored as “Yes” (10 points), “Sometimes” (5 points), or “Not Yet” (0 points).^14^

#### Early Developmental Stages Inventory—Social Emotional

Early developmental stages inventory—social emotional assesses the social and emotional development of children aged 3-66 months through parent report. It covers self-regulation, compliance, communication, autonomy, affect, and social interaction. Items are scored as 0, 5, or 10 points.^15^

### Statistical Analysis

All statistical analyses were performed using SPSS version 26.0 (SPSS Inc., Chicago, Ill, USA). The Kolmogorov-Smirnov test was used to assess the normality of data distribution. Categorical variables were presented as frequencies and percentages (n (%)), whereas continuous variables were reported as mean ± SD for normally distributed data or as median with interquartile range (IQR) for non-normally distributed data. Comparisons of categorical variables were conducted using the chi-square test or Fisher’s exact test, as appropriate. For continuous variables, the Student’s *t*-test was used for normally distributed parameters, and the Mann–Whitney *U*-test was used for non-normally distributed variables. A multiple linear regression analysis was performed to examine predictors of time to diagnosis. A *P*-value of < .05 was considered statistically significant.

## Results

Descriptive information on the sociodemographic characteristics of the participants is presented in Table 1. The majority of children diagnosed with ASD (n = 85, 85%) came from nuclear families, and 41.4% were from families with a single sibling. Among the parents in the sample, 13% (n = 13) reported consanguineous marriages. Of these, 76.9% were first-degree consanguineous marriages (cousin marriages), while 23% were second-degree consanguineous marriages (grandchild marriages).

At the time of birth of children diagnosed with ASD, the mean maternal age was 32.26 years (SD = 6.16; median = 31; range = 21-48 years), while the mean paternal age was 36.42 years (SD = 6.31; median = 35; range = 25-50 years). Among the parents, 25% (n = 25) were categorized as having advanced maternal age, and 30% (n = 30) as having advanced paternal age.

When examining psychiatric history, maternal reports indicated a family history of ASD or pervasive developmental disorders in 10% (n = 10) of cases, delayed milestones in 9% (n = 9), and speech delays in 25% (n = 25). Paternal reports showed a family history of ASD or pervasive developmental disorders in 6% (n = 6) of cases, delayed milestones in 11% (n = 11), and speech delays in 24% (n = 24).

Most parents (n = 74, 74%) reported being the first to notice developmental concerns about their child. This was followed by pediatricians (n = 10, 10%), teachers (n = 5, 5%), child psychiatrists (n = 4, 4%), family physicians (n = 2, 2%), and other relatives (n = 5, 5%).

Parents’ initial concerns were predominantly related to speech delays (63%), social skill delays (63%), stereotypic behaviors/restrictive, repetitive interests (27%), and disruptive behaviors (20%). Most parents (63%, n = 63) expressed their concerns to child psychiatrists, while a smaller proportion (22%, n = 22) sought assistance from developmental pediatricians.

The mean age at which parents first noticed concerns was 23.1 ± 10.94 months (median = 24; range = 5-60 months). The mean age at which an ASD diagnosis was made was 31.54 ± 12.11 months (median = 26; range = 10-64 months). The average time from the initial concern to receiving a diagnosis was 8.44 ± 8.76 months (median = 5; range = 1-46 months).

The mean CARS score for children diagnosed with ASD (n = 100) was 37.83 (SD = 5.41; median = 38; range = 30-52). The clinical characteristics of the participants are presented in Table 2.

According to the CARS scores, 41% (n = 41) of children diagnosed with ASD were classified as having mild ASD, while 59% (n = 59) were classified as having severe ASD. There was no statistically significant difference in age between the mild and severe ASD groups (*P* > .05). However, regarding gender distribution, girls were significantly more likely to be classified in the severe ASD group compared to boys (*P* = .032). No significant differences were observed between the mild and severe ASD groups in terms of socioeconomic status and family structure (*P* > .05).

A developmental assessment was conducted for 85% (n = 85) of children diagnosed with ASD by a child development specialist at XX. According to the developmental test results, children with severe ASD exhibited significantly more delays in communication, personal-social, and social-emotional development compared to those with mild ASD (*P* < .05) (Table 3).

Most parents of children diagnosed with ASD reported that their children had received at least 1 form of intervention, such as medication, alternative therapies, or special education. All children with a prior diagnosis of ASD (n = 23) had received special education interventions, and 30.4% had undergone medication treatment.

In contrast, among children who had not previously been diagnosed with ASD (n = 77), 27.2% (n = 21) had received interventions, and 5.19% (n = 4) had undergone medication treatment. Within the newly diagnosed ASD group, pre-assessment interventions were distributed as follows: 8 children had received speech therapy, 7 had undergone occupational therapy, and 7 had participated in individualized educational interventions. Among those receiving individualized educational support, 3 had engaged in play therapy, 1 had undergone Applied Behavior Analysis (ABA) therapy, and 1 had been supported through the DIR/Floor Time Approach™ approach. A detailed overview of treatment utilization patterns within the ASD sample is provided in Table 4.

In the newly diagnosed ASD group, individuals receiving intervention had significantly lower CARS scores than those who did not (*t* = 2.881, *P* < .01). Moreover, their parents had significantly higher educational levels (mothers: *t* = −2.024, *P* = .030; fathers: *t* = −2.295, *P* = .027). However, a chi-square test revealed no statistically significant association between family socioeconomic status and receiving intervention (*χ*² = 3.029, *P* = .082).

Among children receiving an ASD diagnosis for the first time at ÇÖZGEM (n = 77), 92.2% were referred by child psychiatrists. The initial appointment’s average waiting time was 18.21 ± 10.25 days (median = 15; range = 3-54 days). The diagnostic process took an average of 36.85 ± 19.8 days (median = 30.5; range = 10-123 days), and the average number of sessions required for diagnosis and treatment planning was 5.52 ± 0.97 sessions (median = 5; range = 4-9 sessions) (Table 5).

A multiple linear regression analysis was conducted to investigate the extent to which demographic and clinical factors predict the time until a child receives an ASD diagnosis. The predictors included the child’s age in months, sex, parental education (in years), autism symptom severity (CARS scores), socioeconomic status (SES), and the age at which caregivers first recognized symptoms. The overall regression model was statistically significant, F(7, 61) = 8.63, *P* < .001, and explained approximately 50% of the variance in time to diagnosis, R^2^ = 0.497, Adjusted R^2^ = 0.440.

Among the predictors, child’s age (β = .905, *P* < .001), CARS score (β = −0.240, *P* = .018), and age of first symptom recognition (β = −0.795, *P* < .001) were statistically significant. Other variables—including maternal and paternal education, SES, and sex—did not significantly contribute to the model (*P* > .05).

## Discussion

This study investigated the sociodemographic and clinical characteristics of children diagnosed with ASD who were evaluated at Ankara Etlik ÇÖZGEM between May 2024 and November 2024, as well as to gain a deeper understanding of the services provided through ÇÖZGEM during the period from the emergence of developmental concerns to the ASD diagnosis. ÇÖZGEM’s streamlined diagnostic processes and rapid referral pathways facilitate earlier diagnoses and timely access to interventions. The duration of these processes was considerably shorter compared to global averages. These findings underscore the critical importance of early diagnosis and intervention and emphasize the positive impact of integrated access to health and education services in the multidisciplinary management of ASD.

Studies suggest a marked gender disparity in children with ASD, with a predominance of males. However, reported male-to-female ratios vary considerably across studies. The most recent study by the CDC reported a male-to-female ratio of 4.3:1,^16^ consistent with other research.^17^ Similarly, a recent nationwide study in Türkiye estimated the male-to-female ratio at 4.6:1.^18^ Nevertheless, some community-based studies have reported lower ratios, ranging from 2:1 to 3:1.^19^ In the present study, we identified a male-to-female ratio of 4.9:1, which aligns with findings in the existing literature. This disparity has remained stable over time, though its causes remain unclear. Previous research has suggested that this may be due to camouflaging behaviors in females, differences in symptom presentation, and diagnostic biases. From an etiological perspective, it has also been proposed that females may require a greater genetic or environmental load for ASD to manifest clinically.^19^

Research indicates that parents are typically the first to notice developmental concerns.^20^ The most reported initial concerns among parents involve delays in expressive language. However, limitations in nonverbal communication, such as a lack of eye contact and delays in social behaviors, such as playing with other children, are also frequent concerns. Additionally, some parents identify stereotypic movements, restricted interests, adherence to routines, behavioral problems, and cognitive or motor delays as early signs.^21^ This study revealed that parents’ initial concerns were primarily related to delays in social skills or language, followed by ASD-specific concerns such as stereotypic behaviors and restrictive, repetitive interests. The findings also revealed that greater autism symptom severity, as measured by CARS scores, was associated with a shorter time to diagnosis. This negative association indicates that children presenting with more pronounced or severe symptoms were more likely to receive an earlier diagnosis compared to those with milder clinical presentations. Consistent with previous literature,^22^ this study also found that earlier recognition of symptoms by caregivers significantly reduced diagnostic delay, highlighting the critical role of parental awareness in facilitating timely identification and intervention. As primary observers of early development, parents should be guided to monitor milestones and report concerns to ensure timely access to services.

While global studies report an average age of 17-19.6 months for the emergence of parental concerns,^23^^,^^24^ the authors’ findings indicate a slightly later mean age of 23.1 months in Türkiye. This delay in ASD diagnosis may be influenced by cultural tendencies to adopt a “wait-and-see” approach, concerns about the stigma associated with developmental disorders, and limited awareness among service providers, which can lead to the dismissal of parental concerns. Additionally, socioeconomic disadvantages and cultural beliefs may further contribute to parents’ hesitation in seeking an early diagnosis.^25^^,^^26^ Compared to findings from other studies, the later emergence of parental concerns about ASD in Türkiye may reflect a preference to observe developmental progress before acting. A more in-depth investigation could provide valuable insights into families’ perspectives on “delayed diagnosis,” exploring the issue from both sociological and psychological standpoints. In this study, the mean age of ASD diagnosis was determined to be 31.54 months. A recent study in Türkiye reported a similar mean diagnostic age of 31.06 months,^18^ whereas globally, the diagnostic age ranges between 48 and 54 months.^16^ These findings suggest that children in the authors’ sample accessed diagnosis and intervention more rapidly after parental concern, enabling earlier support. To fully benefit from earlier diagnoses, it is essential to ensure access to adequate and high-quality ASD interventions in Türkiye.

Pediatricians, family physicians, and specialists in other fields play a critical role in the early diagnosis of ASD.^27^ However, studies indicate that primary healthcare providers often fail to identify developmental concerns before parents do.^20^ Research on the diagnostic process reveals that parents consult various professionals during the journey to diagnosis. Recent studies show that parents most frequently share their initial developmental concerns with pediatricians, followed by family physicians, psychologists, and psychiatrists.^20^^,^^21^ This study observed that parents were typically the first to notice developmental delays in their children and that initial consultations most occurred with child psychiatrists, less frequently with other pediatric departments. While the professionals authorized to diagnose ASD vary across countries,^28^ child psychiatry is the designated specialty for ASD diagnosis in Türkiye. Similar to the nationwide screening program implemented in Türkiye,^29^ strategies to enhance the ability of primary care health professionals to recognize ASD symptoms include the integration of routine screening programs into primary care, in-service training programs aimed at increasing healthcare providers’ awareness, and the establishment of streamlined referral pathways that facilitate timely and efficient referrals to ÇÖZGEM across all levels of care.

The waiting time between an initial consultation with a specialist and a final ASD diagnosis is often lengthy. Research shows that parents may wait anywhere from 6 months to 4 years before receiving an official diagnosis for their child.^16^^,^^18^ Many parents attend multiple appointments with different specialists during this time, which can lead to further delays in the diagnostic process. Comprehensive ASD evaluations often require waiting periods ranging from several months to as long as 7 months.^30^ The average time to diagnosis at the multidisciplinary center in the UK was reported to be 375 days.^31^ In contrast, during the first 6 months following the opening of ÇÖZGEM, the average waiting time for an initial appointment was 18.21 days, and the diagnostic process was completed in an average of 36.85 days. These timelines are significantly shorter than global averages. While the relatively short waiting list during the center’s early operational period may have contributed to this difference, this advantage in early diagnosis for children with special needs in Türkiye presents a valuable opportunity. This streamlined diagnostic process could enhance developmental outcomes and adaptive functioning if supported with practical, high-quality, individualized interventions.

Since the etiology of ASD has not been fully elucidated, current treatment approaches focus on alleviating symptoms and enhancing individuals’ social interaction and verbal communication skills.^32^ Research suggests that evidence-based interventions, particularly when initiated early, can reduce the core symptoms of ASD and its associated conditions.^33^ In this study, children who received an ASD diagnosis and initiated intervention had lower symptom severity. Most received speech and occupational therapy. Higher parental education was associated with earlier initiation of these interventions. These findings highlight the importance of increasing autism awareness and promoting evidence-based, individualized interventions.

When compared to multidisciplinary ASD diagnostic models in high-income countries such as the United Kingdom, the United States, Ireland, Australia, and New Zealand, the ÇÖZGEM model demonstrates both similarities and unique features. Internationally, structured evaluations—typically involving pediatricians, psychologists, and speech-language pathologists—are considered the gold standard and are supported by guidelines like NICE (UK) and CDC (US). These teams commonly use standardized tools such as ADOS-2 and ADI-R. Despite their structure, these models face challenges such as long wait times and limited follow-up.^31^^,^^34^^-^^36^ In contrast, the ÇÖZGEM model, embedded in Türkiye’s public health system, offers a streamlined, early-childhood-focused diagnostic process led by child psychiatrists. Assessments comprehensively address the child’s developmental and behavioral profile and parental concerns. The model’s centralized coordination of medical referrals and follow-up care helps ensure timely, individualized interventions. This contrasts with the often siloed and time-intensive procedures reported in systems across the UK and the USA. Furthermore, ÇÖZGEM's reduced wait times and team continuity may support greater diagnostic efficiency and accessibility in middle-income settings.

Nevertheless, there are some limitations specific to the ÇÖZGEM model. Due to resource constraints, standardized observational tools widely used internationally—such as the ADOS-2 and ADI-R—are not routinely implemented at the center. In addition, the reliance on parent-reported measures in certain assessments may limit diagnostic accuracy when not supported by objective observational data. These areas represent important opportunities for further development of the model. Beyond these specific issues, structural limitations are inherent to the multidisciplinary diagnostic framework on which the ÇÖZGEM model is based. The literature frequently highlights several challenges associated with such approaches, including insufficient empirical evidence supporting the effectiveness of specific interventions, conceptual and methodological inconsistencies across disciplines, and the risk of fragmented or eclectic practices lacking theoretical coherence.^37^ Furthermore, implementing such models often requires considerable time and resources, which may limit their sustainability in resource-constrained settings. Differences in professional training and uneven distribution of foundational knowledge—particularly regarding autism or behavior analysis—among team members may further hinder effective collaboration. Acknowledging both the model-specific and systemic limitations is essential for refining the ÇÖZGEM framework to ensure it remains both evidence-based and practically viable. Future studies should include outcome-based comparisons examining diagnostic accuracy, family satisfaction, and longitudinal developmental trajectories to evaluate the model’s scalability and effectiveness across diverse clinical and socioeconomic contexts.

Our study has certain limitations. First, it focuses exclusively on data from children diagnosed with ASD who were referred to ÇÖZGEM. However, ÇÖZGEM provides evaluations for all children aged 0-6 years with special needs, including those with neurodevelopmental disorders and developmental disabilities, who could benefit from early intervention. Expanding the sample size and including cases with different diagnoses could offer a more comprehensive understanding of the patient profile at ÇÖZGEM. Another limitation is the study’s retrospective nature, which may result in incomplete data potentially influencing the findings. Nonetheless, this limitation was minimized through consistent follow-up at a single center and a systematic data recording system. Additionally, this study focuses solely on cases referred to one ÇÖZGEM center, and the findings may not fully represent other centers or the broader population. Furthermore, the assessment tools used relied solely on parent reports, which may introduce subjective bias. While parent-based assessments provide important insights into developmental concerns, they may not fully capture observable behavior. In future studies, integrating structured or semi-structured observational methods—such as ADOS-2 and ADI-R—may enhance diagnostic accuracy and data validity, as the combined use of ADOS and ADI-R is widely considered the “gold standard” for autism diagnosis.^38^

In conclusion, this study raises important questions about the initial referral, diagnosis, and post-diagnostic management processes within the context of ÇÖZGEM. To better understand the strengths and weaknesses of the ASD care system implemented at ÇÖZGEM, longitudinal studies are needed to evaluate the effectiveness of existing care pathways and intervention programs. As this study focuses specifically on the functioning of diagnostic processes, the workflow algorithm was examined to obtain primordial data, which can further guide the development and implementation of this novel approach. In line with the authors’ findings, it is recommended that the multidisciplinary model adopted by ÇÖZGEM be disseminated more broadly to accelerate and standardize the diagnosis of ASD. Health policies should prioritize integrating such structured models into national systems to ensure equitable and timely access to diagnostic and intervention services. Additionally, future research should focus on optimizing diagnostic methodologies and improving communication and coordination between diagnostic teams and service providers to ensure continuity of care. Once diagnostic procedures are standardized and refined within this framework, subsequent studies should address follow-up and rehabilitation services to enhance long-term outcomes. Specialized centers such as ÇÖZGEM can serve as key reference institutions in shaping standardized and accessible diagnostic and care pathways for ASD, ultimately supporting a more sustainable and integrated national developmental and mental health care system.

## Figures and Tables

**Table 1.** Sociodemographic Characteristics of the Autism Spectrum Disorder Sample

	Total (n = 100)
Sex, n (%)	
Girl	17 (17)
Boy	83 (83)
Age (in months) at the time of the clinical visit^a^	40.86 ± 16.92
Family structure, n (%)	
Nuclear family	85 (85)
Extended family	7 (7)
Broken	7 (7)
In institutional care	1 (1)
Consanguineous marriage, n (%)	
No	87 (87)
Yes	13 (13)
Socioeconomic status, n (%)	
Low	14 (14)
Middle	51 (51)
High	35 (35)
Maternal education level (years)^a^	11.99 ± 3.31
Paternal educational level (years)^a^	12.31 ± 3.09
Number of sibling, n (%)	
None	38 (38)
1	41 (41)
2-4	21 (21)
History of psychiatric disorders in siblings, n (%)	
No	84 (84)
Yes	
ASD	8 (8)
Speech disorder	4 (4)
ADHD	3 (3)
SLD	1 (1)

^a^Mean ± SD. ADHD, attention deficit hyperactivity disorder; ASD, autism spectrum disorder; SLD, specific learning disabilities. *Socioeconomic status was classified according to the Hollingshead-Redlich Scale (HRS).

**Table 2.** Clinical Characteristics of the Autism Spectrum Disorder Sample

	Total (n = 100)
Parents’ initial concerns, n (%)	
Speech delay	63 (63)
Delay in social skills	63 (63)
Stereotypic behaviors or restricted interests	27 (27)
Disruptive behavior (hyperactivity, impulsivity, aggression)	20 (20)
Attention deficit	4 (4)
Self-Injurious behavior	3 (3)
Feeding problems	2 (2)
Motor delay	3 (3)
Cognitive delay	2 (2)
Other concerns	17 (17)
History of regression, n (%)	
No	75 (75)
Yes	25 (25)
Age of onset of regression, n (%)	
<12 months	6 (24)
12-36 months	18 (72)
>36 months	1 (4)
Previously reported diagnoses, n (%)	
ASD	23 (23)
Global developmental delay	9 (9)
Speech disorders	6 (6)
Preliminary diagnosis of ADHD	9 (9)
Motor developmental delay	2 (2)
Genetic disorders	3 (3)

ADHD, attention deficit hyperactivity disorder; ASD, autism spectrum disorder.

**Table 3.** Sociodemographic and Clinical Characteristics of Individuals with Autism Spectrum Disorder Based on Symptom Severity

	CARS-Mild (n = 41)	CARS-Severe (n = 59)	*χ*^2/Z/t^	*P*
Age (years), mean ± SD, median (IQR)	43.41 ± 18.25, 38(23)	39.08 ± 15.85 , 40 (23)	1.262^a^	.210
Gender, n (%)				
Boy	38 (92.7)	45 (76.3)	4.618	.032
Girl	3 (7.3)	14 (23.7)		
Socioeconomic status, n (%)				
Low	6 (14.6)	8 (13.6)	1.536	.464
Middle	18 (43.9)	33 (55.9)		
High	17 (41.5)	18 (30.5)		
Family structure, n (%)				
Nuclear family	37 (90.2)	48 (81.4)	2.849	.252
Extended family	3 (7.3)	4 (6.8)		
Seperetad/divorced	1 (2.4)	7 (11.9)		
Diagnosis duration (days)	7.56 ± 9.13	9.05 ± 8.51	0.745^b^	.406
	**CARS-Mild (n = 34)**	**CARS-Severe (n = 51)**		
EGE (fail), n (%)				
Communication	25 (73.5)	48 (94.1)	7.132	.011
Fine motor skills	25 (73.5)	45 (88.2)	3.036	.081
Gross motor skills	19 (55.9)	32 (62.7)	0.400	.527
Problem-solving	20 (58.8)	32 (62.7)	0.132	.716
Personal-social	24 (70.6)	48 (94.1)	8.718	.003
EGE-SE (fail), n (%)	23 (67.6)	48 (94.1)	10.390	.001

Values are mean and standard deviation (SD) ^a^Mann–Whitney U-test, ^b^Student’s *t*-test. ASD, autism spectrum disorder; CARS, childhood autism rating scale; EGE, early developmental stages inventory; EGE-SE, early developmental stages inventory–social emotional; IQR, interquartile range.

**Table 4.** Characteristics of Referral and Treatment Service Utilization in the Autism Spectrum Disorder Sample

	n (%)
First consulted professional specialty (n = 100)	
Child and adolescent psychiatry	63 (63)
Developmental pediatrics	22 (22)
Pediatrics	8 (8)
Pediatric neurology	4 (4)
Family medicine	2 (2)
Psychologist	1 (1)
Treatments received by previously diagnosed with ASD (n = 23)	
Medication	7 (30.4)
Alternative treatments	2 (8.69)
Intervention services	23 (100)
Treatments received by patients with the initial diagnosis of ASD (n = 77)	
Medication	4 (5.19)
Alternative treatments	4 (5.19)
Intervention services	21 (27.2)
Types of intervention services (n = 100)	
Individualized educational program	22 (22)
Group therapy	13 (13)
Speech therapy	20 (20)
Occupational therapy	27 (27)
Exercise and sports programs	3 (3)

ASD, autism spectrum disorder.

**Table 5.** Characteristics of ÇÖZGEM Examinations in Cases with an Initial Autism Spectrum Disorder Diagnosis

	Total (n = 77)
Referring specialty, n (%)	
Child and adolescent psychiatry	71 (71)
Developmental pediatrics	4 (4)
Pediatric neurology	1 (1)
Family physician	1 (1)
Waiting time for first appointment (days)^a^	18.21 ± 10.25
Diagnosis duration (days)^a^	36.85 ± 19.8
Number of diagnosis and evaluation sessions^a^	5.52 ± 0.97
Evaluation of cases by specialties, n (%)	
Child and adolescent psychiatry	77 (100)
Child development specialist	74 (96.1)
Individual service consultant	77 (100)
Occupational therapist	77 (100)
Speech-language therapy specialist	18 (23.3)
Clinical psychologist	4 (5.19)
Clinical social worker	4 (5.19)

^a^Mean ± SD. ASD, autism spectrum disorder; ÇÖZGEM, multidisciplinary child and adolescent mental health center.

## Data Availability

The data that support the findings of this study are available on request from the corresponding author.
